# Knowledge About Chronic Orofacial Pain Among General Dentists of Kermanshah, Iran

**DOI:** 10.2174/1874210601711010221

**Published:** 2017-04-28

**Authors:** Fatemeh Rezaei, Roohollah Sharifi, Hamid R. Shahrezaee, Hamid R. Mozaffari

**Affiliations:** 1 Department of Oral Medicine, School of Dentistry, Kermanshah University of Medical Sciences, Kermanshah, Iran; 2 Department of Endodontics, School of Dentistry, Kermanshah University of Medical Sciences, Kermanshah, Iran; 3 General Dentist, School of Dentistry, Kermanshah University of Medical Sciences, Kermanshah, Iran

**Keywords:** Chronic pain, Orofacial pain, Knowledge, General dentist, Canthomeatal line

## Abstract

**Background and Objective:**

Diagnosis and treatment of chronic orofacial pain are one of the most challenging issues in dentistry. The purpose of this study was to assess the knowledge of general dentists regarding orofacial pain in Kermanshah, Iran.

**Methods:**

This cross-sectional study was conducted in 2016 including general dentists of Kermanshah city. A researcher-designed questionnaire was administered to collect demographic data as well as measuring knowledge of the dentists in four sections including etiology, clinical presentations, physical examination, and treatment of chronic orofacial pain. The questionnaire had acceptable validity (content validity > 0.9) and reliability (intraclass correlation coefficient= 0.857 for test re-test; Cronbach’s alpha= 0.72 for internal consistency). The data were analyzed by the SPSS software (ver. 18.0) using Spearman’s correlation coefficient (*P* < 0.05).

**Results:**

There were 121 male (72.9%) and 45 female (27.1%) dentists with mean (SD) age of 40.55 (8.03) years and mean (SD) practice history of 13.28 (8.43) years. Mean (SD) knowledge score was 10.54 (2.36) (maximum possible score= 15). 48.2% of dentists had good knowledge in overall. 48.2% about etiology, 45.2% about clinical presentations, 36.1% about physical examination, and 7.8% about treatment had good knowledge. Knowledge had direct and significant relationship with age (r = 0.179; *P* = 0.022) and practice history (r = 0.18; *P* = 0.021).

**Conclusion:**

The results showed that the studied dentists did not have enough knowledge about chronic orofacial pain especially in the treatment field. Therefore, it is recommended to implement educational programs to improve their knowledge.

## INTRODUCTION

Orofacial pains are defined as pains in the face or mouth. Facial pain is originated from locations under canthomeatal line, superior to the neck, and anterior to the ears. Oral pain points to pains originated from intra-oral structures [1]. According to the etiology, chronic orofacial pain is categorized into seven categories: neurologic, vascular, musculoskeletal, oral and perioral, psychosomatic, connective tissue disorders, and referral pains. This method of categorization is a practical method for dentists [2].

In a review article By Macfarlane *et al*., the prevalence of orofacial pains was varied between 1% and 48% with the average of 13% considering the definition used [3]. Aggarwal *et al*., in an epidemiologic study reported chronic orofacial pain as 7% in patients aged 18 to 75 years [4]. However, chronic orofacial pains are more common in some special groups. For example, orofacial pains are more common among those who present to dentistry clinics as 53.8% [5] and 44.5% among those who play musical instruments [6].

Considering the relatively high prevalence of orofacial pain in the general population and as it can adversely affect the quality of life of the patients [7-9], prevention, diagnosis, and treatment of such patients are of high importance in improving public health. However, pain evaluation is difficult as pain is a subjective feeling; studies have shown that chronic orofacial pain may be misdiagnosed as pain with dental origin and may lead to incorrect treatment by dentists [10-13].

Prevention, evaluation, diagnosis, treatment, and control of orofacial pains in both forms of stable or recurrent pains are among educational courses provided to dental students and management of such patients is professional responsibility of dentists [14]. However, there are ambiguities regarding treatment of chronic orofacial pain. Orofacial pains are often associated with pain in other parts of the body or general, sleep, social, and movement disorders which complicate diagnosis and treatment. Evidence about treatment is limited and most clinicians prescribe medications based on personal experience or trial and error method [15].

 In addition, information about orofacial pain and temporomandibular joint (TMJ) disorders is not provided uniformly at various universities and in most cases, the education is not reliable and standard [16], and the provided educational materials are spare and insufficient [17]. Most dentists feel that they have less ability to diagnose facial pain and TMJ disorders in comparison to restorative and periodontal treatments [18, 19]. It has been shown that in dentistry centers, standard guidelines for the treatment of chronic orofacial pains are not followed completely and pains related to psychological disorders are not usually treated [20]. Studies have shown that considerable number of dentists is interested in obtaining further information about orofacial pain [21].

Limited number of studies has been conducted to assess the knowledge of dentists about chronic orofacial pains and most of them have emphasized the importance of providing further education in this field [22-24]. The objective of this study was to evaluate the level of awareness of general dentists of Kermanshah, Iran about chronic orofacial pains.

## MATERIALS AND METHODS

In this cross-sectional study, general dentists who were practicing in private offices and general dentistry clinics of Kermanshah city, in 2016, were considered. This study was approved by the Research Deputy of Kermanshah University of Medical Sciences, Kermanshah, Iran. List and addresses of all general dentists (about 220 dentists) in Kermanshah city were received from the Deputy of Treatment of Kermanshah University of Medical Sciences.

The researcher met the dentists in their offices or clinics. After explaining the objectives of the study and assuring them that the data will be kept confidential, if the dentists agreed to participate, he/she was included in the study.

A researcher-designed questionnaire was used to collect the data. It had two sections. The first section included demographic information (age, gender, and practice history). The second section included 15 close-ended multiple choice questions evaluating etiology (3 questions), clinical presentations (4 questions), physical examination (3 questions), and treatment (5 questions) of chronic orofacial pain including burning mouth syndrome, trigeminal neuralgia, TMD, migraine and atypical odontalgia. These questions were chosen from text books and articles [25, 26]. After assessing the reliability/validity, some questions were modified and one question on the field of etiology and one question on physical examination field were eliminated. For correct answer, score 1 and for incorrect/blank answer, no score were assigned. According to this scoring system, the maximum possible score was 15 and the minimum was zero. Scoring system categorized as poor (0-5 score), moderate (6-10 score) and good (11-15 score).

In order to verify the reliability of the questionnaire the questionnaire was delivered to 10 specialist dentists. For assessing the comprehensibility of the questionnaire, the questionnaire was delivered to 5 general dentists. To determine the validity of the questionnaire, content validity index was used which was higher than 0.9 for all questions which were considered to be acceptable. To determine the reliability of the questionnaire, test re-test reliability and internal reliability were used. For test re-test reliability, 30 general dentists filled out the questionnaire two times with two weeks’ time interval. Intra-class correlation coefficient was calculated as 0.857 which was acceptable. To determine internal reliability of the questionnaire, firstly, the Pearson correlation coefficient of each statement of the questionnaire and then the overall score (except of that particular question) were calculated. The Cronbach’s alpha was calculated for questions which was 0.72.

### Statistical Analyses

The data were analyzed by the SPSS software (ver. 18.0). The descriptive indices such as mean and standard deviation were used to express data. To determine the relationship between the level of knowledge with age and practice history, the Spearman’s correlation coefficient was calculated. The significance level was set at 0.05.

## RESULTS

Among 220 dentists who were working in Kermanshah city, Iran, we could find 204 eligible dentists, 166 dentists (81%) including 121 male (72.9%) and 45 female (27.1%) dentists with mean (SD) age of 40.55 (8.03) and mean (SD) practice history of 13.28 (8.43) years filled out the questionnaires. Table **1** presents demographic variables of the samples. To determine normal distribution of the variables, the Kolmogorov-Smirnov test was used (Table **2**). Table **3** presents the frequency of answers to the questions of the questionnaire. The range of correct answers was 42.2% to 91.6%. The most and least frequent correct answers were questions 3 and 1 (etiology), 3 and 4 (clinical presentations), 2 and 3 (physical examination), and 5 and 1 (treatment), respectively. The least frequent correct answers were observed in treatment section.

Table **4** presents mean ±SD knowledge score of the studied dentists regarding chronic orofacial pain. This score was 10.54 ± 2.36 in overall (maximum =15). The score of etiology was 2.37 ± 0.67 (maximum = 3), score of clinical presentation was 3.33 ± 0.69 (maximum = 4), score of physical examination was 2.09 ± 0.82(maximum = 3) and the score of treatment was 2.74 ± 1.23(maximum = 5).

Table **5** shows the knowledge of the dentists regarding chronic orofacial pain based on 4 different categories (etiology, clinical presentations, physical examination and treatment). 48.2% of the dentists had good knowledge regarding chronic orofacial pain (48.2% about etiology, 45.2% about clinical presentations, 36.1% about physical examination, and 7.8% about treatment). 50.6% of the dentists had moderate knowledge regarding chronic orofacial pain (41% about etiology, 54.2% about clinical presentations, 38.6% about physical examination, and 49.4% about treatment). 1.2% of the dentists had poor knowledge regarding chronic orofacial pain (10.8% about etiology, 0.6% about clinical presentations, 25.3% about physical examination, and 42.8% about treatment).

To determine the relationship between overall knowledge and age, the Spearman’s correlation test was used. It was revealed that the correlation was significant and direct (r= 0.179, *P*= 0.022); (Table **6**).

In order to determine the relationship between knowledge level and practice history, the Spearman’s correlation test was used. This showed that a significant and direct association existed between knowledge score and practice history (r= 0.18, *P*= 0.021); (Table **7**).

According to the Spearman’s correlation test, a significant and direct relationship was observed between knowledge and age (r = 0.179, *P* = 0.022) and practice history (r = 0.18, *P* = 0.021).

## DISCUSSION

Dentists often visit patients with chronic orofacial pains, and therefore, it is necessary to have enough knowledge regarding diagnosis and treatment of such patients. Therefore, the current study was carried out with the objective of evaluating the knowledge of general dentists regarding etiology, clinical presentations, physical examination, and treatment of chronic orofacial pains.

The response rate was 81% which is considerable compared to similar studies which reported response rate of 53% to 86% [22, 27, 28]. The current study showed that knowledge of general dentists, approximately half of the dentists (48.2%), about chronic orofacial pain was good and others had moderate (50.6%) or weak (1.2%) knowledge level. Also, limited numbers of dentists had acceptable level of knowledge regarding etiology (48.2%), clinical presentations (45.2%), and physical examination (25.3%). Similarly, Borromeo, in a study evaluating the mechanism of orofacial pain and its treatment, reported that 47% of fourth-year dental students, 58% of final-year dental students, and 48% of the dentists had acceptable level of knowledge [23]. Al-Khotani *et al.*, study showed that level of knowledge of Saudi Arabian and Swedish dentists about orofacial pain was low [24]. In another study, it was reported that newly graduated dentists stated that they had insufficient knowledge regarding orofacial pains [29].

The undesirable level of knowledge among dentists about orofacial pain can be attributed to the lack of educational standards during education regarding this topic which causes ambiguities for dental students [16]. Evaluation of dental schools has shown that educating chronic orofacial pain is insufficient, non-comprehensive, and very limited [17].

Regarding treatment of chronic orofacial pains, only 7.8% of the studied dentists had good knowledge. Level of knowledge of about half of the dentists was moderate and it was undesirable in 42.2% of the dentists. Likewise, a previous study showed undesirable knowledge about the treatment of orofacial pains among dentists [23]. Wirz **et al.** showed that even in academic centers, treatment of chronic orofacial pains is not carried out properly [20]. It has been reported that the role of dentists in the treatment of chronic orofacial pains is unknown and limited [30]. Tegelberg *et al*. reported that the knowledge of general dentists about TMJ disorders was insufficient and the level was lower than specialist dentists [31]. Peters *et al.*, reported that although psychological factors play a role in chronic orofacial pains, treatments provided to patients are insufficient and limited to therapeutic interventions [32]. Alonso *et al*., reported that the lowest level of self-assessment of students about chronic orofacial pain was related to treatment abilities of pain with psychological origins [25].

Nearly three-fourth (76.5%) of the dentists correctly reported brain tumor as one of the etiologic factors of trigeminal neuralgia. Some studies have reported trigeminal neuralgia as one of the manifestations of intracranial tumors such as central nervous system lymphoma [33], meningioma [34], cerebellopontine angle tumors such as cholesteatoma [35], and epidermoid tumor [36]. Therefore, dentists should consider brain tumors as differential diagnoses when facing patients with trigeminal neuralgia.

In the present study, 77.1% of dentists responded that mouth burning syndrome is more common after menopause. This syndrome is very common in middle-aged (40 to 60 years) and post-menopausal women and is associated with sexual hormones [37].

About 83.1% correctly answered the question that “imaginary tooth pain can be felt at a location with tooth loss”. This disorder is limited to dento-alveolar regions which may have teeth or have no teeth [38]. About 80.1% had correct knowledge that “trigeminal neuralgia causes electric shock like pain which is often unilateral”. Trigeminal neuralgia is one of the rare causes of unilateral facial pain and its pain is similar to electric shock and is aggravated by palpation. This pain manifests itself with recurrent daily attacks, although the attacks may not occur for several weeks to months [39, 40].

Most dentists (96.6%) were aware of ‘association between TMJ disorders with clicking sound of the joint and restriction in opening the mouth”. The most important symptom of TMJ disorder is pain and then limitation of the mandibular bone movement which results in difficulty eating and talking. Sound produced from TMJ during movement is another symptom of TMJ disorder.

Many dentists (80.1%) were aware of the importance of examining neck muscles and TMJ in patients with chronic pain. Extra oral examinations in these patients generally include examinations of head and neck. The masseter muscle, scalp and neck muscles, muscular hypertrophy, TMJ movement including crepitus should be evaluated [41].

Only 44% of the dentists had correct knowledge about the use of capsaicin in alleviating local pain of post herpetic neuralgia. Capsaicin is an herbal medicine and its pharmaceutical products with high dosages (8%) are used to manage post herpetic neuralgia and other neuropathic pains [42].

About 81.3% of the dentists correctly answered this statement that “behavioral therapy and medical therapy have role in the treatment of atypical pains”. The best method of treating such patients is a combination of biopsychosocial treatments with anti depressant medications and behavioral therapy [43].

According to the obtained findings, the overall knowledge level had direct significant relationship with age. Increase in the knowledge level of dentists with aging has been shown in several studies [44, 45]. It seems that this relationship is the result of gaining professional experience in older dentists. Also, a significant direct relationship existed between overall knowledge and practice history. In conformity with these findings, Baharvand *et al.*, reported that the knowledge of dentists regarding TMJ disorders increases with longer duration of practice [46].

## CONCLUSION

The current study showed that knowledge of half of the studied dentists was acceptable regarding etiology or clinical presentations. Only one-third and fewer than 10% of the dentists had acceptable level of knowledge regarding physical examination and treatment of chronic orofacial pains. Older dentists and those with longer practice history had higher level of knowledge.

**Limitations:** There was no standard questionnaire for the evaluation of knowledge in general dentists. Therefore, we made researcher-designed questionnaire; it was the most challenging issue in this research. While some of the dentists did not cooperate with us and they did not fill the questionnaire leading to the small sample of dentists, which is a limitation of this study.

**Recommendation:** We recommend that general dentists should have chronic orofacial pain course in academic curriculum for better training to improve their knowledge in this field. Further studies are recommended to be carried out with a larger sample of dentists in the future.

## Figures and Tables

**Table 1:** Measures of central and dispersion tendency of demographic variables based on gender.

		Age	Practice history
Female dentists (N= 45)	Mean	37.36	8.96
	SD	6.91	5.65
Male dentists (N= 121)	Mean	41.77	14.91
	SD	8.13	8.74
Total (N= 166)	Mean	40.55	13.28
	SD	8.03	8.43

SD= standard deviation

**Table 2:** Assessment of normal distribution of the data.

	Age	Practice history	Knowledge
Test statistic	1.183	1.609	1.389
*P* value	0.122	0.011	0.042

*Kolmogorov-Smirnov test

**Table 3:** The results of answers to the questions of the questionnaire.

	Question	Incorrect	Blank (no knowledge)	Correct
		N (%)	N (%)	N (%)
Etiology	One of the causes of trigeminal neuralgia could be brain tumor	25 (15.1%)	14 (8.4%)	127 (76.5%)
	Burning mouth syndrome is more common after menopause	17 (10.2%)	21 (12.7%)	128 (77.1%)
	Orofacial pain could be referral pain from neck muscle disorder	19 (11.4%)	8 (4.8%)	139 (83.1%)
Clinical presentations	Imaginary tooth pain can be produced in a location with tooth loss	12 (7.2%)	16 (9.6%)	138 (83.1%)
	Trigeminal neuralgia produces electric shock like pain which is often unilateral	13 (7.8%)	20 (12%)	133 (80.1%)
	TMJ disorders pain is often associated with clicking sound of the joint and restricted mouth opening	14 (8.4%)	0	152 (91.6%)
	Migraine can cause facial or jaw pain	22 (13.3%)	14 (8.4%)	130 (78.3%)
Physical examination	Local anesthetic tests can be used to evaluate chronic orofacial pain	31 (18.7%)	6 (3.6%)	129 (77.7%)
	Examination of neck muscles and TMJ in patients with chronic pain is important	19 (11.4%)	14 (8.4%)	133 (80.1%)
	Cold spray on muscles has role in examination of chronic pain	48 (28.9%)	33 (19.9%)	85 (51.2%)
Treatment	The first-line treatment for TMJ disorders is diazepam	63 (38%)	33 (19.9%)	70 (42.2%)
	Tricyclic anti depressants can be used for treatment of burning mouth syndrome	55 (33.1%)	32 (19.3%)	79 (47.6%)
	The most frequent medication used for trigeminal neuralgia is carbamazepine	29 (17.5%)	39 (23.5%)	98 (59%)
	Cutaneous capsaicin (red pepper extract) is efficacious for post herpetic neuralgia	36 (21.7%)	57 (34.3%)	73 (44%)
	Behavioral therapy and medical therapy have role for treatment of atypical pains	20 (12%)	11 (6.6%)	135 (81.3%)

TMJ= temporomandibular joint

**Table 4:** Measures of central and dispersion tendency of knowledge of the dentists regarding chronic orofacial pain.

	Overall knowledge	Etiology	Clinical presentations	Physical examination and evaluation	Treatment
Total score of each section	15	3	4	3	5
Mean	10.54	2.37	3.33	2.09	2.74
SD	2.36	0.67	0.69	0.82	1.23
Minimum score	4.00	1.00	1.00	0.00	0.00
Maximum score	15.00	3.00	4.00	3.00	5.00
First quartile	9.00	2.00	3.00	1.00	2.00
Median	10.00	2.00	3.00	2.00	3.00
Third quartile	12	3.00	4.00	3.00	4.00

SD= standard deviation

**Table 5:** Knowledge of the dentists regarding chronic orofacial pain based on 4 categories.

Score	Overall knowledge N (%)	Etiology N (%)	Clinical presentations N (%)	Physical examination &evaluation N (%)	Treatment N (%)
Good	80(48.2)	80(48.2)	75(45.2)	60(36.1)	13(7.8)
Moderate	84(50.6)	68(41)	90(54.2)	64(38.6)	82(49.4)
Poor	2(1.2)	18(10.8)	1(0.6)	42(25.3)	71(42.8)

**Table 6:** Overall knowledge of the dentists regarding chronic orofacial pain based on age categories.

	Poor	Moderate	Good
	N (%)	N (%)	N (%)
< 35 years	1 (2.38%)	26 (61.90%)	15 (35.71%)
35-45 years	1 (1.30%)	37 (48.05%)	39 (50.65%)
> 45 years	0	19 (43.18%)	25 (56.82%)

**Table 7:** Overall knowledge of the dentists regarding chronic orofacial pain based on practice history.

	Poor	Moderate	Good
	N (%)	N (%)	N (%)
< 5 years	0	15 (46.88%)	17 (53.13%)
6-10 years	1 (2.63%)	24 (63.16%)	13 (34.21%)
11-15 years	1 (2.33%)	25 (58.14%)	17 (39.53%)
> 15 years	0	19 (36.54%)	33 (63.46%)
